# Supplementary figures and images for: Relationship between liver dysfunction, lipoprotein concentration and mortality during sepsis
Source: PLoS One. 2022 Aug 22;17(8):e0272352. doi: 10.1371/journal.pone.0272352 (PMC9394828; doi:10.1371/journal.pone.0272352)

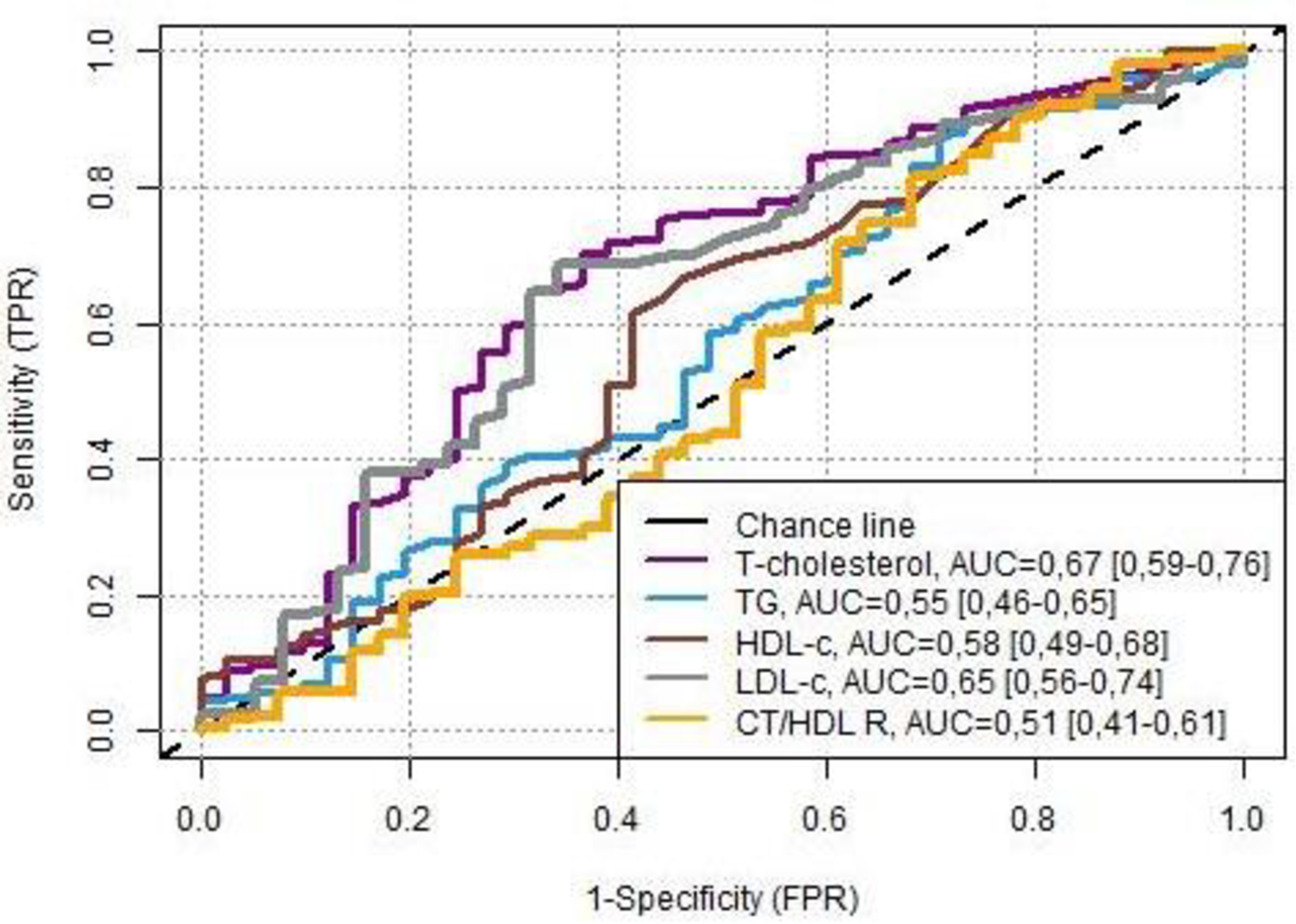

Supplement: S1 Fig — ROC curves were plotted to determine lipid and lipoprotein cutoff values to predict 28-day mortality. T-cholesterol: Total cholesterol; TG: triglycerides; HDL-C: high-density lipoprotein cholesterol; LDL-C: low-density lipoprotein cholesterol. (TIF) [file pone.0272352.s001.tif]
